# The impact of opioid versus non-opioid analgesics on postoperative pain level, quality of life, and outcomes in ventral hernia repair

**DOI:** 10.1007/s10029-024-02968-3

**Published:** 2024-01-31

**Authors:** Ramez Alzatari, Li-Ching Huang, Benjamin K. Poulose

**Affiliations:** 1 Department of Surgery, Center for Abdominal Core Health, The Ohio State University Wexner Medical Center, Columbus, OH USA; 2 Ohio University Heritage College of Osteopathic Medicine—Dublin Campus, Dublin, OH USA; 3 Department of Biostatistics, Vanderbilt University Medical Center, Nashville, TN USA

**Keywords:** Hernia, Opioids, Pain, Outcomes, ACHQC

## Abstract

**Purpose:**

Managing postoperative pain remains a significant challenge in hernia operations. With ventral hernia repair (VHR) being one of the most commonly performed procedures, this study aimed to compare the effectiveness of non-opioid analgesia to opioid-based regimens for postoperative pain management.

**Methods:**

The Abdominal Core Health Quality Collaborative was queried for elective VHR patients between 2019–2022. Subjects prescribed opioid or non-opioid analgesics at discharge were matched using a propensity score. Postoperative Hernia-Related Quality of Life Survey (HerQLes) summary scores, Patient-Reported Outcome Measurement Information System (PROMIS) 3a questionnaire, and clinical outcomes were compared between the two groups.

**Results:**

1,051 patients who underwent VHR met the study criteria. The 2:1 matched demographics were opioids (n = 188) and non-opioids (n = 94) (median age 63, 48% females, 91% white, and 6.5 cm hernia length). Long-term (1-year post-operation) patients’ pain levels were similar between opioids vs non-opioids (median (IQR): 31(31–40) vs. 31(31–40), p = 0.46), and HerQLes summary scores were similar (92(78–100) vs. 90(59–95), p = 0.052).

Clinical short-term (30-days post-operation) outcomes between opioid vs non-opioid patients had similar length-of-stay (1(0–5) vs 2(0–6), P = 0.089), readmissions (3% vs. 1%, P = 0.28), recurrences (0% vs. 0%, P = 1), reoperations (1% vs. 0%, P = 0.55), surgical site infections (3% vs. 7%, P = 0.11), surgical site occurrences (5% vs. 6%, P = 0.57), and surgical site occurrences requiring procedural intervention (3% vs. 6%, P = 0.13). Finally, long-term recurrence rates were similar (12% vs. 12%, P = 1).

**Conclusion:**

Non-opioid postoperative regimens for analgesia are non-inferior to opioids in VHR patients with similar outcomes. Aggressive efforts should be undertaken to reduce opioid use in this population.

## Introduction

Managing postoperative pain remains a significant challenge in hernia operations. With ventral hernia repairs (VHR) being one of the most commonly performed operations [1], much opportunity exists to evaluate non-opioid analgesic regimes for controlling postoperative pain. Pain management is crucial for patients’ postsurgical satisfaction. Previously, pain was described as the Fifth Vital Sign as an initiative to improve pain management [2]. The widespread prescription of opioids for pain increased rapidly in the 1990s, resulting in the ‘first wave’ of opioid overdoses in the United States [3]. Recently, evidence has shown that many General Surgery patients are prescribed opioids upon discharge, with uncertain benefits for pain control [4]. In fact, providers may overprescribe opioids postoperatively, giving patients more pills than necessary [5, 6]. Moreover, chronic use of opioids increases the risk of opioid misuse or abuse [7].

Despite the widespread use of opioids after VHRs, there is limited research on their comparative effectiveness with non-opioids concerning postoperative outcomes. Therefore, this study explored the effectiveness of non-opioid postoperative analgesia compared to opioid-based regimens.

## Methods

### Design overview

This analysis was a retrospective cohort study from a national hernia registry in the United States called the Abdominal Core Health Quality Collaborative (ACHQC). This analysis aimed to compare short- and long-term pain intensity, quality of life (QoL), and clinical postoperative outcomes between those who were prescribed opioids versus non-opioids at discharge after elective VHR.

Hernia-Related Quality-of-Life (HerQLes) survey is a tool used to assess hernia patients’ QoL, with proven reliability. The survey provides a summary score for hernia patients at baseline and after surgery [8]. Patient-Reported Outcome Measurement Information System (PROMIS) 3a is a questionnaire designed by the National Institute of Health to measure pain intensity. The questionnaire has demonstrated both high reliability and validity [9]. As such, HerQLes and PROMIS 3a were used to assess patients’ QoL and pain intensity. All patients at the ACHQC are asked to fill both surveys preoperatively at baseline and postoperatively at 30-day and 1-year follow-up visits.

In this study, we hypothesized that non-opioids would be equally effective to opioids for postoperative outcomes. The Institutional Review Board at The Ohio State University approved the performance of this study.

### Data source

Data were obtained from the ACHQC. The ACHQC is a national hernia registry in the United States that aims for continuous quality improvement for hernia diseases. The ACHQC data collection started in 2013. Surgeons’ and patients’ participation in this registry is voluntary. At the time of analysis, information was available from 438 surgeons in various clinical settings, including academic, private, and private-academic affiliated hospitals. Data collection at the ACHQC is standardized by all surgeons and locations and is obtained in a prospective fashion in real-time [10].

### Population

As this analysis aimed to compare short- and long-term pain intensity, QoL, and clinical postoperative outcomes in the elective setting, the study population included all adult subjects who underwent elective VHRs from 2019 to 2022 within the ACHQC. Inclusion criteria included patients ages 18 or older with an elective VHR during the study period and completed 30-day and 1-year patient-reported HerQLes and PROMIS 3a surveys. Exclusion criteria excluded subjects with missing data on prescribed or non-prescribed opioids and patients with missing patient-reported outcomes (PRO). The PROs refer to the reported outcomes by the patients in the two surveys (HerQLes QoL and PROMIS 3a Pain).

### Comparison groups

This study had two comparison groups based on the analgesics prescribed at discharge. Subjects who were prescribed opioids at discharge were added to the opioids group. In contrast, patients prescribed only non-opioids upon discharge or recommended over-the-counter non-opioids at discharge were added to the non-opioids group.

The exposure variable was the prescription of opioids or non-opioids at discharge after VHR. The opioids group included any prescription of the following regimens: Oxycodone, Oxycodone/Acetaminophen, Hydrocodone/Acetaminophen, Hydromorphone, Codeine, Methadone, Morphine, Meperidine, Tapentadol, Tramadol, or Fentanyl. The non-opioids group included any prescription or over-the-counter recommendation of Acetaminophen, Ibuprofen, Naproxen, Meloxicam, Celecoxib, Pregabalin, or Gabapentin.

### Outcome measures

The primary outcome of this study was the long-term postsurgical outcomes, including PROMIS 3a pain levels, HerQLes QoL summary scores, and pragmatic recurrence rates. The pragmatic recurrence rates were defined as any radiographic, clinical, or patient-reported recurrence element. The long-term outcomes were defined as the outcomes reported at 1-year postoperatively.

The secondary outcome for this analysis was the short-term postoperative outcomes, including PROMIS 3a pain levels, HerQLes QoL summary scores, and clinical outcomes and complications. The short-term outcomes were defined as the outcomes reported at 30-day after surgery. The short-term clinical outcomes and complications included recurrence rates, length of stay, readmissions, recurrences, reoperations, surgical site infections (SSI), surgical site occurrences (SSO), and surgical site occurrences requiring procedural intervention (SSOPI).

SSIs were defined as any wound-related superficial incisional, deep incisional, or organ space infections. SSOs were any incidence of wound cellulitis, non-healing incisional wound, fascial disruption, skin or soft tissue ischemia, skin or soft tissue necrosis, wound serous drainage, wound purulent drainage, chronic sinus drainage, localized stab wound infection, stitch abscess, seroma, infected seroma, hematoma, infected hematoma, exposed biologic mesh, exposed synthetic mesh, contaminated biologic mesh, contaminated synthetic mesh, infected biologic mesh, infected synthetic mesh, mucocutaneous anastomosis disruption, or enterocutaneous fistula. Finally, SSOPIs were any surgical site occurrences that required a follow-up procedural intervention. Procedural interventions included suture excision, wound opening, wound debridement, percutaneous drainage, and partial or complete mesh removal.

### Statistical analysis

Subjects’ demographics, pre-, intra-, and post-operative characteristics were summarized between opioids and non-opioids groups. In this study, continuous variables were summarized by medians and inter-quartile ranges (IQR) and were compared using Wilcoxon rank sum test. The categorical variables were presented as parentages and frequencies and were compared with Person’s Chi-squared test or Fisher’s exact test. HerQLes summary scores were calculated using the following formula: (120−[(20/12)*(sum of response on all 12 questions)]), with a range from 0 to 100 [8]. A higher HerQLes score indicates a better QoL. PROMIS 3a Pain Intensity T scores were calculated according to the guidelines of PROMIS pain intensity instruments [9]. A higher PROMIS 3a score suggests a higher pain intensity, with T scores ranging from 30.7 to 71.8.

The confounding variables in this study include race, sex, body mass index (BMI), wound class, American Society of Anesthesiologists (ASA) class, functional status, sporting status, employment type, surgical approach, intraoperative myofascial release, hernia width, hernia length, pain level at baseline, behavioral health history, prophylactic IV antibiotics, mesh type, age, diabetes, history of chronic opioid use, history of abdominal aortic aneurysm (AAA), and other substance use. To control for these confounding variables, subjects prescribed opioid or non-opioid analgesics were matched using propensity score methods. The confounding variables were adjusted using propensity score matching (PSM) with a ratio of 2 to 1 (opioids to non-opioids). The nearest neighbor without a caliper-matching approach was used. Due to the low missing rate (< 3%) in the confounding variables, only complete cases were included in the PSM analysis. Statistical significance was set at p-values < 0.05. A standardized mean difference (SMD) plot was created to assess the result of the propensity score match with an a priori cutoff of < 2 deemed as an acceptable balance. All analyses were conducted using R version 4.1.

## Results

### Population

Of those who had an elective VHR, 1,051 met the inclusion and exclusion criteria. The study population median (IQR) age was 61 (51–68) years and predominantly white 91%, non-diabetic 82%, non-smoker 95%, had myofascial release 62%, had open surgical approach 74%, independent functional status 97%, with ASA class of 2 or 3 92%, and with median hernia width of 8cm. The cohort was equally split between males and females 50% and with hypertension 51%. The comprehensive demographics are listed in Table 1.

**Table 1:** Comparison groups demographics and clinical characteristics before and after propensity score matching

Variable		Unmatched			Matched		
		Opioids N = 948	Non-opioids N = 103	*P*-value	Opioids N = 188	Non-opioids N = 94	P-value
Basic demographics and clinical characteristics							
Age range (18–90)	Median (IQR)	61 (50–68)	62 (52–68)	0.60	62 (52–69)	63 (55–68)	0.80
Gender, N (%)	Male	475 (50%)	54 (52%)	0.65	99 (53%)	47 (50%)	0.67
	Female	473 (50%)	49 (48%)		89 (47%)	47 (50%)	
BMI	Median (IQR)	32 (28–35)	30 (27–34)	0.063	31 (27–35)	31 (27–34)	0.59
Race, N (%)	White	865 (91%)	94 (91%)	0.99	170 (90%)	86 (91%)	0.77
	Non-white	83 (9%)	9 (9%)		18 (10%)	8 (9%)	
Hypertension, N (%)	Yes	491 (52%)	43 (42%)	0.053	96 (51%)	41 (44%)	0.24
	No	457 (48%)	60 (58%)		92 (49%)	53 (56%)	
Diabetes, N (%)	Yes	176 (19%)	16 (16%)	0.45	39 (21%)	16 (17%)	0.46
	No	772 (81%)	87 (84%)		149 (79%)	78 (83%)	
Current Smoker, N (%)	Yes	54 (6%)	3 (3%)	0.24	10 (5%)	3 (3%)	0.42
	No	894 (94%)	100 (97%)		178 (95%)	91 (97%)	
History of AAA, N (%)	Yes	9 (1%)	0 (0%)	0.32	0 (0%)	0 (0%)	1
	No	939 (99%)	103 (100%)		188 (100%)	94 (100%)	
Operative details							
ASA Class, N (%)	1	42 (4%)	16 (16%)	< 0.001	13 (7%)	16 (17%)	0.028
	2	263 (28%)	27 (26%)		71 (38%)	26 (28%)	
	3	622 (66%)	53 (51%)		102 (54%)	52 (55%)	
	4	15 (2%)	0 (0%)		2 (1%)	0 (0%)	
Hernia Grade, N (%)	1	244 (26%)	38 (37%)	0.007	61 (32%)	32 (34%)	0.39
	2	540 (57%)	42 (41%)		94 (50%)	40 (43%)	
	3	164 (17%)	23 (22%)		33 (18%)	22 (23%)	
Surgical approach, N (%)	Open	691 (73%)	88 (85%)	0.005	154 (82%)	80 (85%)	0.96
	MIS (Laparoscopic/Robotic)	249 (26%)	13 (13%)		24 (16%)	6 (12%)	
	MIS convert to open	8 (1%)	2 (2%)		4 (2%)	2 (2%)	
Myofascial release	Yes	601 (63%)	47 (46%)	< 0.001	84 (45%)	46 (49%)	0.50
	No	347 (37%)	56 (54%)		104 (55%)	48 (51%)	
Hernia width (cm)	Median (IQR)	8 (3–15)	5 (1.2–13)	0.002	5 (2–13)	6.5 (1.5–14)	0.70
Hernia length (cm)	Median (IQR)	15 (3–23)	8 (1.5–20)	< 0.001	5 (2–20)	10.5 (2–20)	0.67
Mesh used, N (%)	Yes	875 (92%)	81 (79%)	< 0.001	146 (78%)	73 (78%)	1
	No	73 (8%)	22 (21%)		42 (22%)	21 (22%)	
Mesh type, N (%)	Biological tissue-derived	7 (1%)	1 (1%)	0.45	1 (1%)	1 (1%)	0.88
	Permanent synthetic	865 (99%)	79 (98%)		143 (98%)	71 (97%)	
	Resorbable synthetic	3 (0%)	1 (1%)		2 (1%)	1 (1%)	
Prophylactic IV antibiotics	Yes	931 (98%)	102 (99%)	0.54	186 (99%)	93 (99%)	1
	No	17 (2%)	1 (1%)		2 (1%)	1 (1%)	
Wound status, N (%)	Clean	784 (83%)	80 (78%)	0.087	155 (82%)	72 (77%)	0.36
	Clean-contaminated	86 (9%)	17 (17%)		27 (14%)	16 (17%)	
	Contaminated	72 (8%)	6 (6%)		6 (3%)	6 (6%)	
	Dirty/Infected	6 (1%)	0 (0%)		0 (0%)	0 (0%)	
Activity level							
Functional status, N (%)	Independent	915 (97%)	101 (98%)	0.36	186 (99%)	92 (98%)	0.78
	Partially dependent	4 (0%)	1 (1%)		1 (1%)	1 (1%)	
	Totally dependent	0 (0%)	0 (0%)		0 (0%)	0 (0%)	
	Unknown	29 (3%)	1 (1%)		1 (1%)	1 (1%)	
Sporting activity, N (%)	Unknown	667 (70%)	78 (76%)	0.64	144 (77%)	70 (74%)	0.90
	None	110 (12%)	8 (8%)		18 (10%)	8 (9%)	
	Sporadic	61 (6%)	8 (8%)		14 (7%)	7 (7%)	
	Moderate	48 (5%)	4 (4%)		6 (3%)	4 (4%)	
	Intense	62 (7%)	5 (5%)		6 (3%)	5 (5%)	
Employment type, N (%)	Unknown (Defaulted)	553 (58%)	71 (69%)	0.21	129 (69%)	63 (67%)	0.82
	No employment	191 (20%)	16 (16%)		32 (17%)	16 (17%)	
	Desk-based labor/Rest	75 (8%)	9 (9%)		19 (10%)	8 (9%)	
	Light physical labor	70 (7%)	5 (5%)		5 (3%)	5 (5%)	
	Moderate physical labor	32 (3%)	2 (2%)		3 (2%)	2 (2%)	
	Heavy or very heavy physical labor	27 (3%)	0 (0%)		0 (0%)	0 (0%)	
Pain management and opioids history							
TAP block, N (%)	Yes	30 (4%)	1 (2%)	0.30	6 (5%)	1 (2%)	0.32
	No	667 (96%)	62 (98%)		124 (95%)	58 (98%)	
Epidural use, N (%)	Yes	20 (3%)	1 (2%)	0.55	5 (4%)	1 (2%)	0.43
	No	677 (97%)	62 (98%)		125 (96%)	58 (98%)	
Recent opioid use (within 30 days), N (%)	Yes	34 (4%)	2 (2%)	0.38	1 (1%)	1 (1%)	0.62
	No	914 (96%)	101 (98%)		187 (99%)	93 (99%)	
Chronic use of provider prescribed opioids (> 90 days), N (%)	Yes	34 (4%)	2 (2%)	0.38	4 (2%)	2 (2%)	1
	No	914 (96%)	101 (98%)		184 (98%)	92 (98%)	
Chronic use of non-provider prescribed opioids (> 90 days), N (%)	Yes	3 (0%)	0 (0%)	0.57	0 (0%)	0 (0%)	1
	No	945 (100%)	103 (100%)		188 (100%)	94 (100%)	
Other substance use, N (%)	Yes	52 (5%)	2 (2%)	0.12	6 (3%)	2 (2%)	0.61
	No	896 (95%)	101 (98%)		182 (97%)	92 (98%)	
Psychiatric history							
MDD, N (%)	Yes	69 (7%)	8 (8%)	0.86	12 (6%)	7 (7%)	0.74
	No	879 (93%)	95 (92%)		176 (94%)	87 (93%)	
Anxiety disorder, N (%)	Yes	78 (8%)	9 (9%)	0.86	10 (5%)	7 (7%)	0.48
	No	870 (92%)	94 (91%)		178 (95%)	87 (93%)	
Other psychiatric disorders	Yes	19 (2%)	1 (1%)	0.47	1 (1%)	1 (1%)	0.62
	No	929 (98%)	102 (99%)		187 (99%)	93 (99%)	

*BMI* body mass index, *IQR* inter-quartile range, *ASA* American Society of Anesthesiology, *TAP* transversus abdominis plane, *MDD* major depressive disorder

### Before propensity score matching

The unmatched population included 948 patients who were prescribed opioids and 103 patients who were prescribed non-opioids at discharge. The opioid population before matching was more complex than the non-opioid population. The opioids patients had higher ASA classes (Class 3: 66% vs. 51%, P < 0.001), higher intraoperative myofascial release (63% vs. 46%, P < 0.001), larger hernia length (15 vs. 8 cm, P < 0.001), larger hernia width (8 vs. 5 cm, P = 0.002), and had more mesh usage (92% vs. 79%, P < 0.001). The basic demographics, comorbidities, hernia characteristics, and operative details are shown in Table 1.

### After propensity score matching

**Figure. 1:**
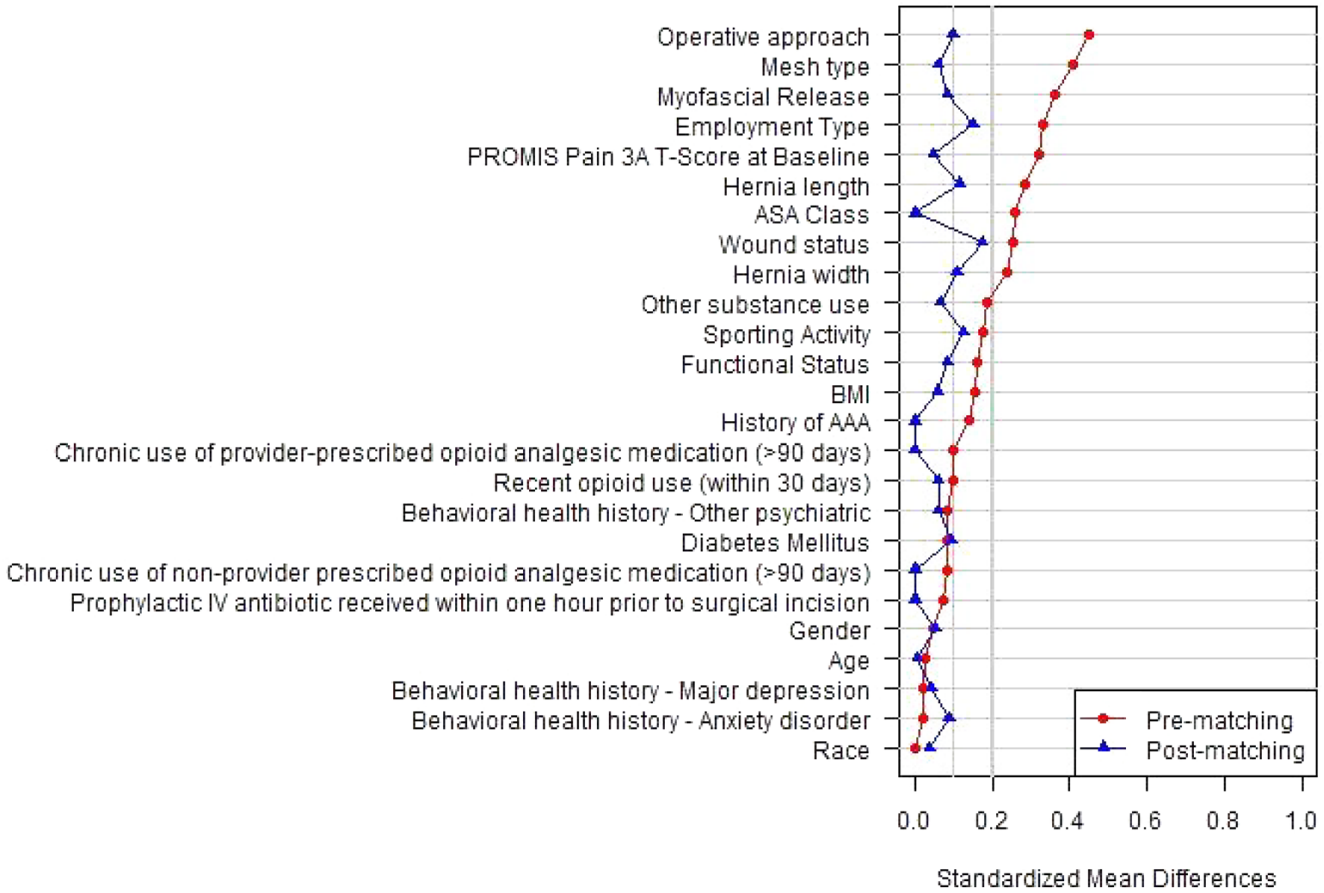
Standardized Mean Difference (SMD) Plot Show Balanced opioids and non-opioids Groups Before and After Propensity Score Matching: Matching was performed on the following variables shown on the *Y*-axis, including race, sex, body mass index (BMI), wound class, American Society of Anesthesiologists (ASA) class, functional status, sporting status, employment type, surgical approach, intraoperative myofascial release, hernia width, hernia length, pain level at baseline, positive behavioral health history, prophylactic antibiotics, mesh type, age, diabetes, history of chronic opioid use, history of abdominal aortic aneurysm (AAA), and other substance use. All covariates met the SMD cutoff of < 0.2, achieving the intended balance

Twenty-five patients, including 9 non-opioid and 16 opioid patients with missing values in adjusted variables, were excluded before PSM. The remaining 932 opioid patients were considered for PSM, with the remaining 94 patients in the non-opioids group. As a result, 188 patients in the opioids group were propensity score-matched to 94 patients in the non-opioids group, as shown in Table 1. The SMD for the basic characteristics before and after propensity score matching are shown in (Fig. 1). While Fig. 1 represents the SMD, the matched variables’ P-values are represented in Table 1. All covariates achieved an SMD < 0.2 and twenty of twenty-five variables achieved an SMD cutoff < 0.1, overall indicating excellent balance after matching.

#### Pain intensity

The long-term PROMIS 3a T scores were similar between opioids vs non-opioids (median (IQR): 31(31–40) vs. 31(31–40), p = 0.46). However, the short-term non-opioids group had lower pain intensity (median (IQR): 40(31–46) vs. 44(40–49), P = 0.012), as shown in Table 2.

**Table 2:** PROMIS Pain 3a scores before and after propensity score matching

	Unmatched			Matched		
	Opioids N = 948	Non-opioids N = 103	P-Value	Opioids N = 188	Non-opioids N = 94	P-value
PROMIS 3a pain score at baseline, median (IQR)	45 (36–52)	44 (31–49)	0.002	40 (31–49)	42 (31–49)	0.66
PROMIS 3a pain score at 30 day, median (IQR)	46 (40–52)	40 (31–46)	< 0.001	44 (40–49)	40 (31–46)	0.012
PROMIS 3a pain score at 30 day (change from baseline), median (IQR)	0.0 (−5.6–9.5)	0.0 (−5.9–5.9)	0.058	1.2 (−3.2–9.5)	0.0 (−5.9–5.9)	0.011
PROMIS 3a pain score at 1 year, median (IQR)	31 (31–44)	31 (31–40)	0.19	31 (31–40)	31 (31–40)	0.46
PROMIS 3a pain score at 1 year (change from baseline), median (IQR)	−5.8 (−13.1–0.0)	−3.3 (−12.8–0.0)	0.079	−3.3 (−12.8–0.0)	−2.9 (−10.6–0.0)	0.57

*PROMIS* Patient-Reported Outcome Measurement Information System, *IQR* inter-quartile range

#### Quality of life

The long-term HerQLes summary scores between opioids vs non-opioids were similar (median (IQR): 92(78–100) vs. 90(59–95), p = 0.052). Additionally, short-term QoL was similar between opioids and non-opioids (median (IQR): 68(43–87) vs. 72(40–92), P = 0.34), as detailed in Table 3.

**Table 3:** HerQLes quality of life summary scores before and after propensity score matching

	Unmatched			Matched		
	Opioids N = 948	Non-opioids N = 103	P-Value	Opioids N = 188	Non-opioids N = 94	P-value
HerQLes Summary score at baseline, median (IQR)	43 (22–70)	55 (33–80)	0.003	60 (35–85)	58 (32–81)	0.56
HerQLes Summary score at 30 day, median (IQR)	58 (37–82)	72 (40–92)	0.002	68 (43–87)	72 (40–92)	0.34
HerQLes Summary score at 30 day (change from baseline), median (IQR)	8.3 (−5.0–26.7)	6.7 (−8.3–30.0)	0.6	5.0 (−8.8–23.8)	4.2 (−8.3—29.2)	0.53
HerQLes Summary score at 1 year, median (IQR)	87 (63–95)	90 (62–96)	0.39	92 (78–100)	90 (59–95)	0.052
HerQLes Summary score at 1 year (change from baseline), median (IQR)	28.3 (8.3–50.8)	21.7 (0.0–39.2)	0.005	23.3 (3.3–46.7)	18.3 (0.0–36.2)	0.17

*HerQLes* Hernia-Related Quality of Life Survey, *IQR* inter-quartile range

#### Clinical postoperative outcomes and complications

The long-term pragmatic recurrence rates between opioids vs non-opioids were similar (12% vs. 12%, P = 1). Short-term outcomes between opioids vs non-opioids patients had similar length of stay (median(IQR): 1(0–5) vs. 2(0–6), P = 0.089), readmission (3% vs. 1%, P = 0.28), recurrence (0% vs. 0%, P = 1), reoperation (1% vs. 0%, P = 0.55), surgical site infections (3% vs. 7%, P = 0.11), surgical site occurrences (5% vs. 6%, P = 0.57), and surgical site occurrences requiring procedural intervention (3% vs. 6%, P = 0.13). More detailed postoperative outcomes are shown in Table 4.

**Table 4:** Clinical outcomes and complications before and after propensity score matching

	Unmatched			Matched		
	Opioids N = 948	Non-opioids N = 103	P-Value	Opioids N = 188	Non-opioids N = 94	P-value
Length of stay (days): median (IQR)	2 (0–5)	2 (0–6)	0.57	1 (0–5)	2 (0–6)	0.089
Readmission (30-day)	5%	1%	0.075	3%	1%	0.28
Recurrence (30-day)	0%	0%	1	0%	0%	1
Reoperation (30-day)	2%	1%	0.4	1%	0%	0.55
Surgical site infections (30-day)	4%	7%	0.18	3%	7%	0.11
Surgical site occurrences (30-day)	9%	6%	0.23	5%	6%	0.57
Surgical site occurrences requiring procedural intervention (30-day)	5%	6%	0.63	3%	6%	0.13
Pragmatic recurrence rate (1-year)	12%	11%	0.60	12%	12%	1

*IQR* inter-quartile range

## Discussion

This study compared the effectiveness of opioid and non-opioid analgesic regimens after elective VHR. It has been shown that non-opioids can be used successfully for postoperative pain control in this surgical population. Both opioid and non-opioid regimens had similar long- and short-term postoperative pain intensity, QoL, and clinical outcomes and complications. The only exception was the non-opioids at 30-day post-operation, where they reported lower pain scores. While the population’s demographics, clinical characteristics, and pain at baseline were matched using a propensity score, it is challenging to explain the reason behind non-opioid patients reporting lower pain scores at the 30-days when compared to opioid patients after surgery. However, it can be that these subjects simply reported low pain levels during their in-patient stay, resulting in non-opioid prescriptions at discharge for pain management rather than opioids. Additionally, studies have shown that opioid use postoperatively is associated with poor pain outcomes and functional impairment [11, 12]. As such, non-opioid analgesics should be considered non-inferior and a viable option for postsurgical pain control in patients after VHR, especially for patients with a high risk of opioid dependence.

While postoperative opioid use is associated with poor pain outcomes and functional impairment, there are many reasons to mitigate opioid postoperative prescriptions and to consider non-opioids more often. Opioid reduction in hernia operations can be significantly impactful as VHR is one of the most commonly performed surgical procedures in the United States, with over 600,000 procedures performed annually [1]. Additionally, the opioid epidemic has been described as the leading cause of overdose deaths in the United States [3]. In fact, one in sixteen patients with opioid prescriptions after surgery will become chronically dependent on opioids [13]. As a result, many studies investigated ways to reduce opioid use in postoperative patients. For example, Ciampa et al. [14] discussed the importance of patient pain management education and shared decision-making with hernia patients in reducing the average opioid prescription size from 12.29 to 6.80 pills. Similarly, VHR patients receiving guideline-based opioid prescriptions were sent home with lower opioid dosages [15]. Furthermore, over one-third of inguinal hernia patients required no opioids for postoperative pain management [16]. Many studies addressed the appropriate number of opioid pills after hernia repairs. For example, Michigan-OPEN recommended 0 to 10 tablets [17], Overton et al. recommended prescribing 0 to 15 pills [18], and Hill et al. advised using 15 tablets [19]. As these studies are moving in the right direction to reduce opioid prescription in hernia patients, no studies have compared any analgesic alternatives in hernia patients. The originality of this study comes from being the first study to compare the effectiveness of non-opioid with opioid regimens for postsurgical pain management in elective VHR.

This study adds significant data about the feasibility of using non-opioid analgesics as a successful alternative to opioids in controlling postoperative pain. The current notion that opioids are the only successful method to control pain may not be accurate. We believe non-opioids should always be considered while discharging VHR patients when clinically appropriate. In many clinical practices, opioids are prescribed as the primary agent to control pain, with non-opioids to augment the opioids’ effects on pain. However, we may need to start thinking about opioids and non-opioids as equal agents when controlling pain in ventral hernia patients, as this study showed. Furthermore, we may need to start thinking about non-opioids as the primary regimen for pain and augment with opioids if non-opioids are not successful in managing pain in a particular patient. This line of thinking is a worthwhile endeavor to reduce opioid use and its consequences. Additional studies are needed for other classes of hernias and to compare individual classes of non-opioid regimens.

This analysis has several limitations. Both HerQLes and PROMIS 3a scores are reported outcomes filled by patients and can be subject to personal bias by VHR patients. However, both surveys were well studied [8, 9]. This analysis is limited to elective VHR cases without emergency case consideration, and future studies are needed for emergency VHRs. The ACHQC data collection started in 2013. However, only data starting from 2019 was reported, as 2019 is when the ACHQC started opioid data collection, which may create bias. However, the ACHQC data collection is standardized at all locations and undergoes audits and quality assurance processes, ensuring maximal data quality [10]. Propensity score matching, although helpful in minimizing bias using non-randomized data, is still limited by bias introduced by unmeasured/unobserved factors. The exposure groups could not be balanced on these types of factors. Finally, the database does not capture the postoperative morphine equivalents that were used in the opioids group.

## Conclusion

In conclusion, we investigated using non-opioids to control postoperative VHR pain. Non-opioid regimens were non-inferior in managing postoperative pain when compared to opioids. Non-opioid prescription should always be considered for VHR patients at discharge when clinically appropriate.

## Data Availability

This research study was conducted retrospectively from de-identified data from the Abdominal Core Health Quality Collaborative (ACHQC). The data is available upon request and approval through the ACHQC.

## References

[1] Schlosser KA, Renshaw SM, Tamer RM, Strassels SA, Poulose BK (2022) Ventral hernia repair: an increasing burden affecting abdominal core health. Hernia 27(2):415–421. 10.1007/s10029-022-02707-6 36571666 10.1007/s10029-022-02707-6

[2] Scher C, Meador L, Van Cleave JH (2018) Reid MC (2018) Moving beyond pain as the fifth vital sign and patient satisfaction scores to improve pain care in the 21st century. Pain Manag Nurs 19(2):125–129. 10.1016/j.pmn.2017.10.010 29249620 10.1016/j.pmn.2017.10.010 PMC5878703

[3] Centers for Disease Control and Prevention (CDC) (2011) Vital signs: overdoses of prescription opioid pain relievers—United States, 1999—2008. MMWR Morb Mortal Wkly Rep 60(43):1487–1492 22048730

[4] Do U, El-Kefraoui C, Pook M, Balvardi S, Barone N, Nguyen-Powanda P, Lee L, Baldini G, Feldman LS, Fiore JF Jr (2022) McGill Better Opioid Prescribing Collaboration; Alhashemi M, Antoun A, Barkun JS, Brecht KM, Chaudhury PK, Deckelbaum D, Di Lena E, Dumitra S, Elhaj H, Fata P, Fleiszer D, Fried GM, Grushka J, Kaneva P, Khwaja K, Lapointe-Gagner M, McKendy KM, Meguerditchian AN, Meterissian SH, Montgomery H, Rajabiyazdi F, Safa N, Touma N, Tremblay F (2022) Feasibility of prospectively comparing opioid analgesia with opioid-free analgesia after outpatient general surgery: a pilot randomized clinical trial. JAMA Netw Open 5(7):e2221430. 10.1001/jamanetworkopen.2022.21430 35849399 10.1001/jamanetworkopen.2022.21430 PMC9294998

[5] Pruitt LCC, Swords DS, Russell KW Rollins MD, Skarda DE (2019) Prescription vs. consumption: opioid overprescription to children after common surgical procedures. J Pediatr Surg 54(11):2195–2199. 10.1016/j.jpedsurg.2019.04.013 31072677

[6] Fafaj A, Zolin SJ, Rossetti N, Thomas JD, Horne CM, Petro CC, Krpata DM, Prabhu AS, Rosenblatt S, Rosen MJ (2020) Patient-reported opioid use after open abdominal wall reconstruction: how low can we go? Surgery 168(1):141–146. 10.1016/j.surg.2020.04.008 32499045 10.1016/j.surg.2020.04.008

[7] Fishbain DA, Cole B, Lewis J, Rosomoff HL, Rosomoff RS (2008) What percentage of chronic nonmalignant pain patients exposed to chronic opioid analgesic therapy develop abuse/addiction and/or aberrant drug-related behaviors? A structured evidence-based review. Pain Med 9(4):444–459. 10.1111/j.1526-4637.2007.00370.x 18489635 10.1111/j.1526-4637.2007.00370.x

[8] Krpata DM, Schmotzer BJ, Flocke S, Jin J, Blatnik JA, Ermlich B, Novitsky YW, Rosen MJ (2012) Design and initial implementation of HerQLes: a hernia-related quality-of-life survey to assess abdominal wall function. J Am Coll Surg 215(5):635–642. 10.1016/j.jamcollsurg.2012.06.412 22867715 10.1016/j.jamcollsurg.2012.06.412

[9] Revicki DA, Chen WH, Harnam N, Cook KF, Amtmann D, Callahan LF, Jensen MP, Keefe FJ (2009) Development and psychometric analysis of the PROMIS pain behavior item bank. Pain 146(1–2):158–169. 10.1016/j.pain.2009.07.029 19683873 10.1016/j.pain.2009.07.029 PMC2775487

[10] Poulose BK, Roll S, Murphy JW, Matthews BD, Todd Heniford B, Voeller G, Hope WW, Goldblatt MI, Adrales GL, Rosen MJ (2016) Design and implementation of the Americas Hernia Society Quality Collaborative (AHSQC): improving value in hernia care. Hernia 20(2):177–189. 10.1007/s10029-016-1477-7 26936373 10.1007/s10029-016-1477-7

[11] Sjøgren P, Grønbæk M, Peuckmann V, Ekholm O (2010) A population-based cohort study on chronic pain: the role of opioids. Clin J Pain 26(9):763–769. 10.1097/AJP.0b013e3181f15daf 20842015 10.1097/AJP.0b013e3181f15daf

[12] Turner JA, Shortreed SM, Saunders KW, LeResche L, Von Korff M (2016) Association of levels of opioid use with pain and activity interference among patients initiating chronic opioid therapy: a longitudinal study. Pain 157(4):849–857. 10.1097/j.pain.0000000000000452 26785321 10.1097/j.pain.0000000000000452 PMC4939796

[13] Brummett CM, Waljee JF, Goesling J Moser S, Lin P, Englesbe MJ, Bohnert ASB, Kheterpal S, Nallamothu BK (2017) New persistent opioid use after minor and major surgical procedures in US adults [published correction appears in JAMA Surg. 2019 Mar 1;154(3):272]. JAMA Surg 152(6):e170504. 10.1001/jamasurg.2017.0504 10.1001/jamasurg.2017.0504 PMC7050825 28403427

[14] Ciampa ML, Liang J, O’Hara TA, Joel CL, Sherman WE (2023) Shared decision-making for postoperative opioid prescribing and preoperative pain management education decreases excess opioid burden. Surg Endosc 37(3):2253–2259. 10.1007/s00464-022-09464-8 35918546 10.1007/s00464-022-09464-8

[15] Lindros SH, Warren JA, Carbonell AM 2nd, Cobb WS 4th, Floyd SB (2023) Implementation of a patient-tailored opioid prescribing guideline in ventral hernia surgery. J Surg Res 282:109–117. 10.1016/j.jss.2022.09.021 36270120 10.1016/j.jss.2022.09.021

[16] Knight AW, Habermann EB, Ubl DS, Zielinski MD, Thiels CA (2019) Opioid utilization in minimally invasive versus open inguinal hernia repair. Surgery 166(5):752–757. 10.1016/j.surg.2019.05.012 31229314 10.1016/j.surg.2019.05.012

[17] Michigan-OPEN (2023) Opioids prescribing recommendations. https://michigan-open.org/prescribing-recommendations/. Accessed 13 Aug 2023

[18] Overton HN, Hanna MN, Bruhn WE, Hutfless S, Bicket MC, Makary MA (2018) Opioid-prescribing guidelines for common surgical procedures: an expert panel consensus. J Am Coll Surg 227(4):411–418. 10.1016/j.jamcollsurg.2018.07.659 30118896 10.1016/j.jamcollsurg.2018.07.659 PMC6353661

[19] Hill MV, McMahon ML, Stucke RS, Barth RJ (2017) Wide variation and excessive dosage of opioid prescriptions for common general surgical procedures. Ann Surg 265(4):709–714. 10.1097/SLA.0000000000001993 27631771 10.1097/SLA.0000000000001993

